# Community networks of sport and physical activity promotion: an analysis of structural properties and conditions of cooperation

**DOI:** 10.1186/s12889-022-14383-3

**Published:** 2022-10-26

**Authors:** Laura Wolbring, Steffen Christian Ekkehard Schmidt, Claudia Niessner, Alexander Woll, Hagen Wäsche

**Affiliations:** Institute of Sports and Sports Science, Karlsruhe Institute of Technology (KIT), Engler-Bunte-Ring 15, 76131 Karlsruhe, Germany

**Keywords:** Health promotion, Interorganizational cooperation, Social network analysis, Sport development

## Abstract

**Background:**

The importance of intersectoral cooperation networks among community organizations located in people’s immediate environments in addressing population health problems such as physical inactivity has come into focus in recent years. To date, there is limited evidence on how and why such networks emerge. Therefore, the aims of this study were (a) to analyze the *structural properties* and (b) to identify the *conditions of cooperation* in interorganizational community networks of sport and physical activity promotion.

**Methods:**

Survey data on cooperative relationships and organizational attributes of sports and physical activity providers as well as sports administrating organizations in two community networks located in urban districts in southern Germany were collected (Network I: n = 133 organizations; Network II: n = 50 organizations). Two quantitative descriptive procedures – network analysis and stochastic analyses of network modeling (exponential random graphs) – were applied.

**Results:**

Similar structures and conditions of cooperation were found in the networks (e.g. low density, centralization). The community sports administrations had the most central positions in both networks. Exponential random graph modeling showed that cooperation took place more frequently in triangular structures (closure effect) and revolved around a few central actors (preferential attachment effect). Organizations from different sectors cooperated more often than organizations from the same sector (heterophily effect).

**Conclusion:**

The study provided valid and robust findings on significant mechanisms and conditions of interorganizational cooperation in community networks focused on sport and physical activity promotion. Based on the results, implications for the development and most efficient governance of these networks can be derived.

**Supplementary Information:**

The online version contains supplementary material available at 10.1186/s12889-022-14383-3.

## Background

The importance of sport and physical activity (PA) in the prevention of non-communicable diseases has been widely demonstrated [1]. However, recent studies have shown that PA levels worldwide are low [2, 3]. In Germany, for example, PA recommendations were only met by a quarter of children and adolescents [4] while about 40% of German adults show insufficient PA behavior [3]. Due to increased mortality rates and health care costs [5, 6], physical inactivity represents a key social and economic challenge.

Individual behavioral interventions have proven insufficient to promote sport and PA at the population level [7, 8]. Instead, interventions aimed at changing systems while taking into account the social and physical environment in which people live have received increasing attention [9, 10]. The World Health Organization not only calls for the provision of individual PA programs and opportunities but also for the development of active systems [11]. In this context, the focus lies on intersectoral cooperation between relevant stakeholders and improved governance to enable social and environmental development and ensure sustainable sport and PA promotion.

To address the rather low PA levels of the German population, the German Federal Ministry of Health published the National Recommendations for PA and PA Promotion (NRPP) [12]. These emphasize the need for PA promotion especially in community settings. While there are projects to implement the promotion of PA on a community level [13, 14], a systematic and nationwide implementation of the NRPP on a policy level is deficient. Therefore, stakeholders call for sport and PA promotion to be given a higher priority on the political agenda, and for better networking of relevant actors including the community level [15].

The community is seen as a central setting in which sport and PA promotion should be implemented since this is the place where people live, learn, work, commute, and exercise [16]. Bauman et al. [17] found that the existence of PA opportunities and recreational facilities in a person’s immediate environment is of great significance when it comes to sport and PA participation. Thus, organizations providing and coordinating sports and PA at the community level and their cooperation efforts play an important role [18, 19]. In particular, the relevance of educational institutions, community departments, sports clubs, and recreational facilities is emphasized [20]. This is because they can provide better access to sports and PA and break down barriers to active transportation through coordinated cooperation and exchange [16]. These not only offer formal sports and PA programs but also provide spaces for informal sports, such as football fields, green spaces, or schoolyards.

The rationale for intersectoral cooperation is that public health challenges, such as physical inactivity, are very complex and multifaceted and therefore cannot be solved by single actors and organizations [21, 22]. In addition, public funding in this area is scarce, which means that cooperation is essential in terms of uniting and sharing resources, information, and expertise [23–26]. Ideas and solutions can be developed jointly and organizational capacity can be built together to address public health problems efficiently and effectively [22, 27, 28]. Researchers have repeatedly emphasized that the health sector is not capable of solving these challenges on its own [29]. Therefore, it is necessary for organizations from various sectors to work together to draw on diverse resources and capabilities and to unite different perspectives on a problem that enables them to reach shared goals [10, 26, 30]. However, intersectoral cooperation is also accompanied by challenges such as increased bureaucracy, differing agendas pursued by individual organizations, and increased time requirements [31]. To address these challenges and to increase network effectiveness, systematic network coordination and management is essential [30].

The present study is based on three interrelated theoretical approaches: (1) systems thinking and the socio-ecological model; (2) network research; and (3) resource dependence theory. First, the concept of systems thinking [32, 33] seeks to go beyond linear and simplistic views of complex phenomena and emphasizes the complexity of social life [34]. It focuses on the diverse interactions of different components and facets of public health problems [35]. According to systems thinking, it is important to understand the different structures that shape people’s lives as well as the interrelations between those structures. This is a necessary prerequisite to be able to transform systems that affect the public’s health. In line with this, the socio-ecological model assumes that, beyond individual action, human behavior is shaped by existing structures at various levels and environments. To change people’s PA behavior, the relevant environments, such as the organizational level, must be addressed [16, 36, 37]. Second, network research is based on the concept of systems thinking and adopts a relational perspective. That means phenomena of interest are explained by reference to their underlying structures. Accordingly, organizations are embedded in social structures and do not act in isolation but in mutual dependence. Thus, it is not the individual organizations that are the unit of analysis but their relationships to each other [38–40]. Social network analysis (SNA) enables the identification of strengths and opportunities for improvement by analyzing the structure of relationships and interactions between organizations from diverse sectors pursuing different goals [41, 42]. Third, according to resource dependence theory [43], organizations build cooperation to gain access to resources they do not possess themselves and thereby try to minimize risks and uncertainties [44–46]. Often, relationships are established with particularly popular organizations, which play a central role in the network and thus have a strong influence on network processes [47]. In Barabási’s terms, this phenomenon is known as scale-free networks [48].

SNA has been increasingly used in many areas of public health research to visualize and examine interorganizational cooperation [22, 41, 49] addressing, for example, tobacco control [50], child abuse prevention [51], HIV services [52], health policy [53], mental health services [54], and the physical and social health of senior citizens [55].

Studies on cooperation networks of organizations engaged in sport and PA promotion show rather heterogeneous results [31], both in terms of network characteristics and in terms of the predictors of cooperation. While some networks have a moderate to high density with a variety of realized relationships [56–58], other networks are rather fragmented with low levels of cooperation [18, 19, 59, 60]. In some networks, cooperation is characterized by centralization of a few actors that hold by far the highest number of cooperative ties or act as gatekeepers [56, 58, 60, 61], whereas in other networks the relationships between the organizations are evenly distributed and represent a decentralized network [19, 59, 62]. There are also contrasting results regarding the conditions of cooperation. In some studies using SNA, organizations in the same sector cooperate more often with each other, indicating homophily as a mechanism of cooperative tie formation [59, 63]. However, other network studies have found that organizations from different sectors are more likely to establish a relationship, indicating heterophily as a mechanism of cooperative tie formation [18, 56, 60]. An effect frequently observed is that cooperation in these networks takes place in triangles [18, 63], i.e. in group-like structures characterized by mutual support and trust [64–66].

The different findings can be attributed to various reasons: (1) Some of the networks studied not only included organizations based in the community but also organizations operating on higher administrative levels, such as the national, state, or county level [56–58, 61–63]. (2) Some of the networks are formally organized with a clear structure and leadership [20, 56–58, 63], while others emerged unplanned without systematic governance [18, 59, 62]. (3) Not all networks focus exclusively on sport and PA promotion but more generally on healthy lifestyles [57, 59] or more specifically on active transportation [67], resulting in different actor constellations. (4) The majority of studies used descriptive methods of network analysis [20, 57–59, 61, 62, 68], while only a small proportion used stochastic methods to uncover the mechanisms and conditions of network emergence [18, 19, 56, 63, 67]. As a result, very few general conclusions concerning the processes and partnerships necessary to build and develop interorganizational community networks promoting sport and PA can be drawn to date. However, to ensure sustainable sport and PA promotion by strengthening partnerships, creating synergetic effects, and building capacity, it is essential to understand how these networks function.

Therefore, the aims of this study are (a) to analyze the *structural properties* and (b) to identify the *conditions of cooperation* in interorganizational community networks of sport and PA promotion. This study will add to the body of knowledge by moving beyond the description of network structures and focusing on organizational and structural predictors of interorganizational cooperation for sport and PA promotion on the community level. For this purpose, interorganizational networks of sport and PA promotion will be analyzed to identify how these networks are structured, how cooperation comes into being, and whether similar characteristics and mechanisms can be found. The findings can help to provide a better understanding of how community networks work and might help to uncover starting points for network development and effective network governance.

## Methods

### Sampling and procedure

The study took place in Germany, where sports and PA are principally organized in non-profit sports clubs as well as in the commercial fitness centers and gyms of the private sector. The public sector includes mainly kindergartens, schools, and universities. Moreover, the public sector comprises community departments and administrations that play important roles due to funding as well as financial and material support for many sports and PA providers of the public and non-profit sector.

For our analysis, we used existing data on two networks in two different communities in southern Germany, which had been collected in earlier studies [69–71]. Hence, we performed a secondary analysis. Both networks were not formally established but emerged unplanned without a formal or strategic goal, also defined as serendipitous networks among organizations [72]. The organizations were connected by contributing to the total of opportunities for sports, PA, and recreational activities and were identified through the subsequent procedure. The data were collected by us following a comprehensive and systematic search to identify relevant community sports and PA providers as well as sports administrating and coordinating organizations. Based on a broad understanding of sports, not only traditional and commercial sports facilities and providers, such as sports clubs and gyms, but also institutions offering sports and PA programs of any form, such as schools, kindergartens, universities, social institutions, churches, and care facilities, were included. In addition, organizations that assumed superordinate, administrative, and advisory functions concerning sport and PA were taken into account. Data were collected in both networks through a standardized online questionnaire that was emailed to the identified organizations. To increase the response rate, follow-up was conducted by email or telephone if no response was received.

Network I was surveyed at the level of an entire city. The city had around 80,000 inhabitants. Initial data was collected in January and February 2012. Network II was surveyed at the level of a city district. The district had about 20,000 inhabitants, with the whole city having around 300,000. Data collection took place from May to August 2017.

### Measures

#### Organizational characteristics

Organizations were divided into three sectors to test for homophily or heterophily as mechanisms of cooperative tie formation: the public sector (e.g. community administrations, schools, kindergartens, universities), the private sector (e.g. gyms, yoga studios, physical therapy practices), and the non-profit sector (e.g. sports clubs, social and church organizations). Additionally, all organizations were divided into for-profit (private sector) and non-profit (public and non-profit sector) organizations to test for activity effects based on for-profit orientation. Organizations in Network II were additionally asked whether they owned a sports facility located in the corresponding city district, as such a resource might trigger cooperation in the sense of resource dependence theory [43].

#### Network characteristics

The survey of cooperative relationships was based on previous studies [63, 73]. Participants were given a list of all identified community sports and PA providers as well as sports administrating and coordinating organizations of the respective setting and were asked to indicate with whom they cooperate and what this cooperation looks like. Up to ten organizations with which a cooperative tie existed could be indicated. If organizations cooperated with more than ten other organizations, they were asked to only name the most important ten. In Network I, the cooperation had to be classified in each case according to one of the following four categories: exchange of information, informal cooperation (loose cooperation to achieve common goals), formal cooperation (close cooperation in a team to achieve common goals), and partnership (close cooperation over a longer period in different projects). In Network II, participants were asked to differentiate between the following cooperation types: exchange of information, exchange of personnel, cooperation on offers, and use of sports facilities. Detailed information on the questionnaires used for data collection can be found in Additional file 1.

As in previous studies [50, 63, 67, 74], both networks were dichotomized so that organizations were considered to be linked if they indicated any type of cooperation. In this way, there is either a cooperative link or not and data can be compared more easily.

### Data analysis

#### Descriptive analysis

To examine structural network properties, Ucinet Version 6.721 [75] and Visone Version 2.19 [76] were used. The networks were visualized and the following parameters were calculated.

On the network level, density (ratio of all realized relationships to the maximum number of possible relationships in the network), average degree (average number of relationships of the organizations), average distance (average shortest path between a set of two organizations), and degree centralization (extent to which all relationships of the network are organized around a few central organizations) were calculated. On the organizational (node) level, degree centrality (CD) (number of relationships with other organizations) and betweenness centrality (CB) scores (extent to which an organization acts as a bridge between two organizations that are not directly connected) were calculated for each organization. More information on the network parameters used can be found in Borgatti et al. [40].

#### Exponential random graph models

To identify conditions and mechanisms of cooperation, we estimated exponential random graph models (ERGMs). ERGMs allow predictions about the probability of cooperative tie emergence between any two network organizations based on the properties of the network and organizational characteristics. They can provide evidence about rules for how and why certain relationships and their combinations occur while assuming that observations, such as network ties, are not independent [77]. Networks are assumed to consist of smaller micro-configurations that describe the structure of the network. ERGMs allow conclusions to be drawn about whether certain micro-configurations in a network are observed more or less frequently than would be expected by chance. A distinction is made between structural network effects, which arise from within the network due to dynamics of self-organization, and attributive network effects, which are due to the characteristics of the organizations [78–80].

We used Markov chain Monte Carlo methods to estimate the parameters of the ERGMs. Model building took place in three stages using R Version 4.0.5 [81]. Model 1 was a null model with no predictors, in model 2 we added the node attributes, and in model 3 the structural predictors were added.

*Model 1*. A simple random graph model, which contains only a single term, the edges term (number of relationships), and predicts the probability of a relationship in the network [82].

*Model 2*. Organizational characteristics were added to the model as node attributes to test their influence on cooperative tie formation. For-profit orientation and owning a sports facility (only in Network II) were added as dichotomous variables. Sector (public, private, non-profit) was included as a factor capturing a differential homophily effect, i.e. to test whether organizations tend to cooperate with organizations from the same sector or not.

*Model 3*. In this model, structural predictors were added to identify structural network effects. For this purpose, the three terms geometrically weighted edgewise shared partner distribution (GWESP), geometrically weighted degree distribution (GWDegree), and geometrically weighted dyad-wise shared partner distribution (GWDSP) were included [83–86]. These account for complex structures and dependency patterns in networks. The GWESP term was added to account for patterns of transitivity within the networks. It captures the tendency of two organizations that share a cooperative tie to form complete triangles with other organizations in the network. The GWDegree term captures the likelihood of organizations with higher degrees (relationships) forming cooperative ties with one another. The GWDSP term was included to measure the structural equivalence of the networks. It captures the tendency of dyads (a set of two unconnected organizations) to have shared neighbors.

To examine model fit, we compared Akaike information criterion (AIC) scores throughout model building. Smaller AIC scores indicate better fit. To check whether the final models (model 3 including attribute and structural predictors) represent the observed networks well, more in-depth goodness-of-fit tests were performed. For this purpose, the distribution of degree (proportion of nodes with respective number of ties), edgewise-shared partners (proportion of edges that show multiple triangulation), triad census (proportion of closed triangles), and minimum geodesic distance (proportion of dyads with the respective shortest path between them) in the observed networks were compared to the distribution of the same characteristics in networks simulated based on the final ERGMs [77, 87].

## Results

### Identified networks

Regarding Network I, a total of 213 relevant actors were identified, of which 159 responded to the survey (74.6% response). Cooperative activity was identified in 104 organizations. Since binary data only provide information about whether a relationship exists or not and cooperation is inherently reciprocal, any cooperative tie from one organization to another can always be regarded as undirected and symmetrical [40]. Thus, respective ties were reconstructed by symmetrization and included in the network for those organizations that had not participated in the survey themselves (n = 29). Therefore, the final cooperation Network I consisted of 133 organizations.

Out of 72 identified actors for Network II, 39 (54.2% response) participated in the survey. 28 organizations indicated cooperative relationships with other organizations and 22 additional organizations could be reconstructed through symmetrization. Thus, the final cooperation Network II consisted of 50 organizations.

In both networks, mainly kindergartens and private sports providers were among the organizations showing no cooperative activity. In Network I, also church institutions as well as nursing homes indicated few or no cooperative ties to other organizations.

### Structural properties

Organizational characteristics are displayed in Table 1. The proportion of public, private, and non-profit organizations was similar in both networks. Non-profit organizations made up the majority, followed by public organizations, with private organizations being the least represented. In Network I, the percentage of non-profit organizations was slightly higher than in Network II. On the other hand, organizations from the public and private sectors were less represented in Network I compared to Network II.

**Table 1:** Organizational characteristics of Network I and Network II

	Network I (*n* = 133)	Network II (*n* = 50)
**Sector**		
Public	32 (24.06%)	18 (36%)
Private	9 (6.77%)	5 (10%)
Non-profit	92 (69.17%)	27 (54%)
**For-profit orientation**		
Yes	9 (6.77%)	5 (10%)
No	124 (93.23%)	45 (90%)
**Possession of a sports facility**		
Yes	-	34 (6%)
No	-	16 (32%)

Data are represented in *n* (%)

**Fig. 1:**
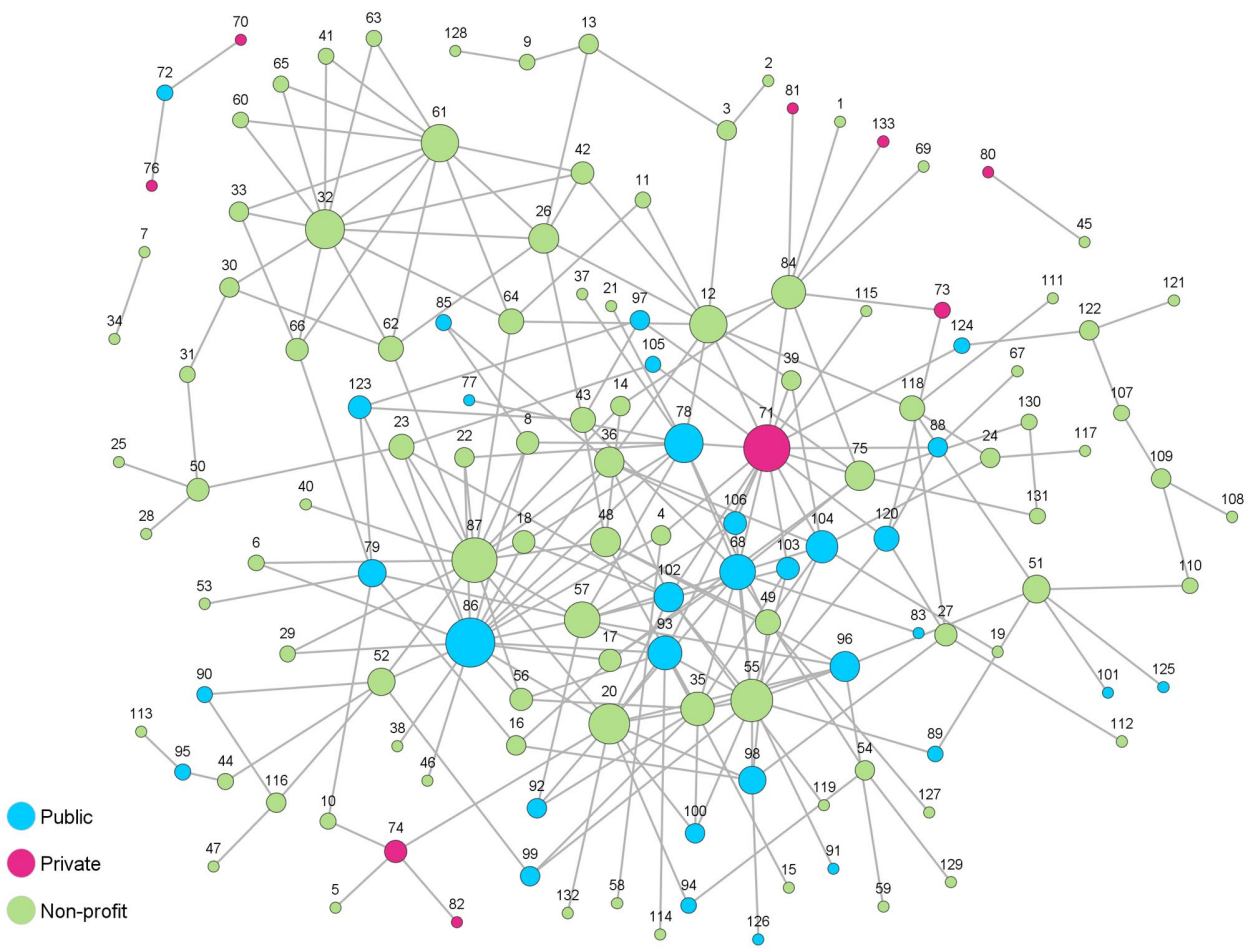
Network I (*n* = 133), ties between nodes indicate cooperation, node color represents sector affiliation, node size represents CD score (number of cooperative ties to other organizations)

**Fig. 2:**
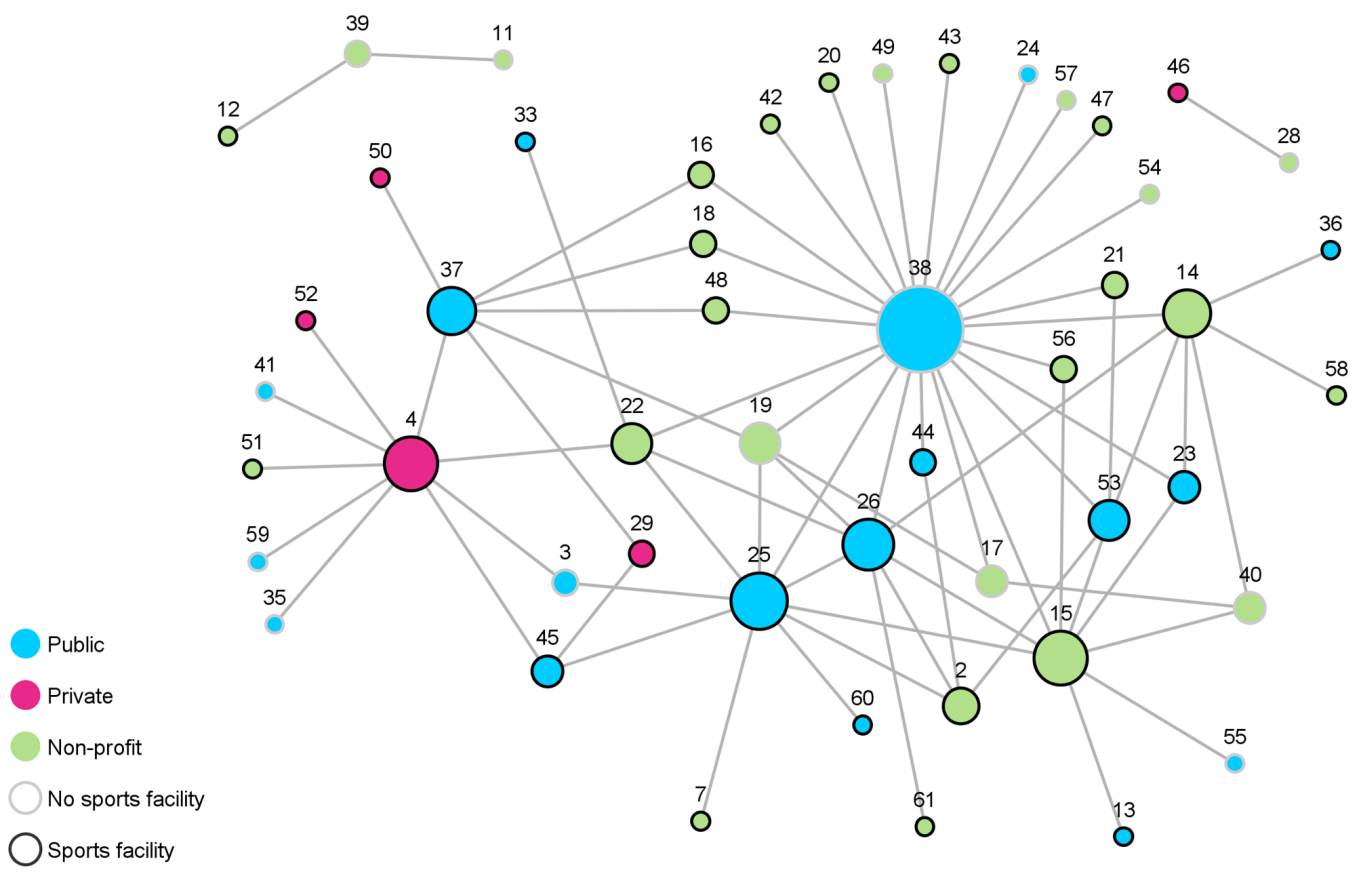
Network II (*n* = 50), ties between nodes indicate cooperation, node color represents sector affiliation, node boarder color represents possession of sports facility, node size represents CD score (number of cooperative ties to other organizations)

Between the 133 organizations of Network I (Fig. 1), 480 cooperative ties were realized. The average degree was 3.61 with a standard deviation (SD) of 3.57, indicating that one organization cooperated on average with three to four other organizations. In Network II (Fig. 2), 148 cooperative relationships existed between the 50 network members and the average degree was 2.96 (SD = 3.75). The density of Network I was 0.03, which means that 3% of all possible ties are realized. Network II also had a relatively low density with 0.06. The minimum number of relationships held by an organization in both networks was one. The maximum number of relationships was 19 in Network I and 23 in Network II. Network II was more centralized, with a degree centralization of 0.43 compared to Network I with a value of 0.12. Organizations were connected to all other actors in the network (average distance) through an average of 3.87 (SD = 1.38) ties in Network I and 2.70 (SD = 0.94) in Network II.

The CD and CB scores of the ten highest scoring organizations are displayed in Table 2. Based on the number of cooperative ties, the community sports administrations (Network I: node 86; Network II: node 38) occupy the most central position in both networks. Other central actors in Network I are a company that manages the community swimming pools (node 71), an association of all community sports clubs (node 87), and two sports clubs (node 55 and 20). In Network II, other central actors are a school (node 25), a private-sector health center (node 4), a sports club (node 15), and another school (node 26). It is noticeable that, in Network II, the community sports administration holds by far the most cooperative relationships (node 38, CD = 23) while the school in position 2 (node 25, CD = 10) has less than half as many connections. In Network I, on the other hand, the degree distribution seems to decrease linearly.

**Table 2:** Normalized CD and CB scores of the ten highest scoring organizations (Network I and II)

Network I					
Node ID	Type of organization	Sector	No. of ties	CD	CB
86	Community sports administration	Public	19	0.14	0.14
71	Administration of community swimming pools	Private	17	9.13	0.18
87	Association of community sports clubs	Non-profit	16	0.12	0.14
55	Local sports club	Non-profit	14	0.11	0.08
20	Local sports club	Non-profit	13	0.10	0.12
78	University sports provider	Public	12	0.09	0.07
32	Local sports club	Non-profit	12	0.09	0.05
12	Local life-saving organization	Non-profit	11	0.08	0.15
61	Local sports club	Non-profit	11	0.08	0.04
68	Health insurance company	Public	10	0.08	0.08
**Network II**					
Node ID	Type of organization	Sector	No. of ties	CD	CB
38	Community sports administration	Public	23	0.47	0.46
25	Public school	Public	10	0.20	0.16
4	Private health center	Private	9	0.18	0.19
15	Local sports club	Non-profit	9	0.18	0.10
26	Public school	Public	8	0.16	0.08
14	Educational outdoor park	Non-profit	7	0.14	0.08
37	University institute for sports	Public	7	0.14	0.09
19	Local sports club	Non-profit	5	0.10	0.04
22	Local sports club	Non-profit	5	0.10	0.16
53	Public school	Public	5	0.10	0.01

In Network I, the company that manages the community swimming pools (node 71) occupies the most central role regarding CB, indicating a powerful role in terms of information control within the network. It is followed by a local life-saving organization (node 12), the community sports administration (node 86), the association of all community sports clubs (node 87), and a sports club (node 20), which also held a high score concerning CD. In Network II, the community sports administration (node 38) not only holds the highest CD but also the highest CB score, which emphasizes its important role concerning the flow of information within the network. It is followed by the private health center (node 4), a sports club (node 22), the school (node 25), and another sports club (node 15), which also held a high score concerning CD.

### ERGMs

The results of the ERGMs for Network I and Network II are displayed in Table 3. Below, we only refer to the final model 3 including the attribute and structural predictors.

**Table 3:** Exponential random graph models for Network I and Network II

Network I									
	Model 1: Null model			Model 2: Attribute predictors			Model 3: Attribute and structural predictors		
Parameters	b (SE)	OR	CI	b (SE)	OR	CI	b (SE)	OR	CI
**Edges**	-3.57 (0.07)***	0.03	0.02–0.03	-3.31 (0.10)***	0.04	0.03–0.04	-3.24 (0.16)***	0.04	0.03–0.05
**Attribute predictors**									
*Homophily*									
Public sector				-0.58 (0.33)	0.56	0.29–1.08	-0.73 (0.35)*	0.48	0.24–0.95
Private sector				0.53 (1.09)	1.70	0.20-14.44	0.51 (1.17)	1.66	0.17–16.59
Non-profit sector				-0.42 (0.14)**	0.65	0.49–0.87	-0.36 (0.13)**	0.70	0.54–0.89
*Activity*									
For-profit orientation				-0.39 (0.22)	0.68	0.44–1.04	-0.31 (0.17)	0.74	0.53–1.02
**Structural predictors**									
GWESP							0.48 (0.08)***	1.62	1.38–1.89
GWDegree							-0.94 (0.21)***	0.39	0.26–0.59
**Model fit**									
AIC	2203			2201			2120		
**Network II**									
	Model 1: Null model			Model 2: Attribute predictors			Model 3: Attribute and structural predictors		
Parameters	b (SE)	OR	CI	b (SE)	OR	CI	b (SE)	OR	CI
**Edges**	-2.74 (0.12)***	0.06	0.05–0.08	-2.35 (0.29)***	0.10	0.05–0.17	-2.30 (0.38)***	0.10	0.05–0.21
**Attribute predictors**									
*Homophily*									
Public sector				-0.32 (0.36)	0.72	0.35–1.47	-0.64 (0.42)	0.53	0.23–1.20
Private sector				1.22 (1.22)	3.40	0.31–37.14	1.30 (1.31)	3.65	0.28–47.58
Non-profit sector				-1.43 (0.39)***	0.24	0.11–0.52	-1.38 (0.44)**	0.25	0.11–0.60
*Activity*									
For-profit orientation				-0.55 (0.34)	0.58	0.29–1.13	-0.55 (0.30)	0.58	0.32–1.04
Sports facility				0.02 (0.19)	1.02	0.70–1.47	0.01 (0.15)	1.01	0.76–1.35
**Structural predictors**									
GWESP							0.36 (0.18)*	1.44	1.02–2.03
GWDegree							-0.84 (0.42)*	0.43	0.19–0.98
**Model fit**									
AIC	560.8			553			543		

*p < 0.05; **p < 0.01; ***p < 0.001. Abbreviations: b = estimate; SE = standard error; OR = odds ratio; CI = 95% confidence interval

Both models show some similarities regarding significant mechanisms of cooperative tie emergence. Concerning the attribute predictors, the estimate for the non-profit sector is significant and negative in both networks. This indicates that organizations from the non-profit sector cooperate with each other less frequently than would be expected by chance, which is also referred to as heterophily. For-profit orientation was not associated with higher cooperative activity in either network. Similarly, owning a sports facility (data only available Network II) did not influence cooperative activity.

With regard to structural network effects, we found a positive tendency for transitivity (GWESP) in both networks, meaning that collaborative ties are more likely to occur in triangular clusters. The GWDegree estimate is significant and negative in both models, which can be interpreted as a preferential attachment effect [88], indicating that cooperation revolves around a few central organizations in both networks. The GWDSP parameter, indicating a tendency of dyads to have shared neighbors, was excluded in both models due to poor convergence.

The two networks differ concerning the cooperation of organizations from the public sector. While there is a heterophily effect for public sector organizations in Network I, meaning that public sector organizations are less likely than chance to cooperate, this effect is not significant in Network II.

### Model fit

**Fig. 3:**
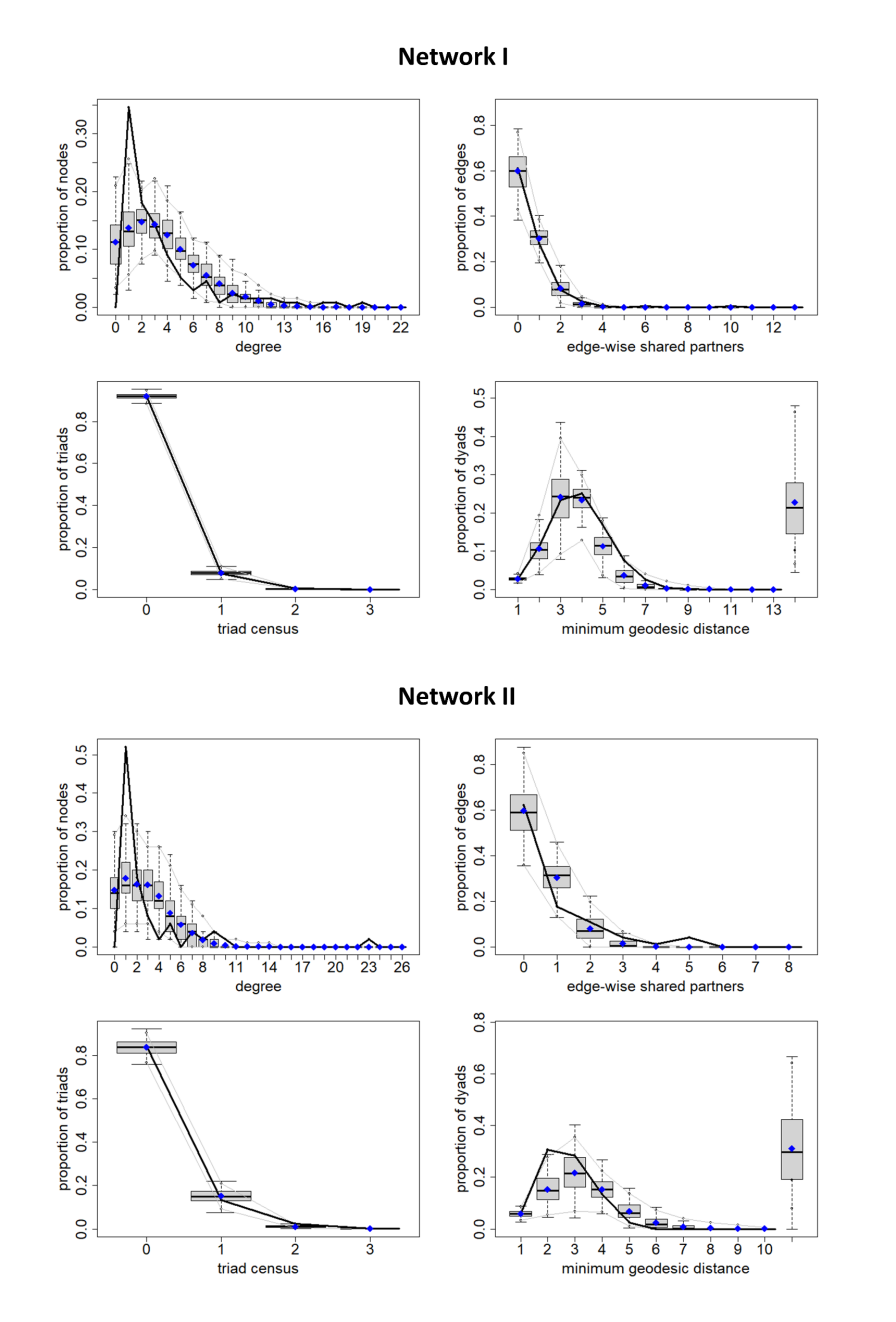
Goodness-of-fit for final Network I model and final Network II model. Gray 95% confidence interval displays proportion of nodes with the respective characteristic in the simulated networks based on the final ERGM, black line represents proportion of nodes with the respective characteristic in the observed networks

When comparing the AIC scores, the final model (model 3) had the best fit in both networks (see Table 3). Goodness-of-fit statistics are displayed in Fig. 3 and show satisfactory model fit for the final models. The gray 95% confidence interval displays the proportion of nodes with the respective characteristic (degree, edgewise-shared partners, triad census, or minimum geodesic distance) in the simulated networks based on the final ERGM (model 3). The black line represents the proportion of nodes with the respective characteristic in the observed networks.

## Discussion

The purpose of this study was to analyze interorganizational cooperation in community networks focused on sports and PA. By investigating two cooperation networks of community sports and PA providers as well as sports administrating and coordinating organizations, we identified structures and predictors that facilitate cooperation and which enable us to uncover starting points for strategic network development and network management in sport and PA promotion.

First, we examined the structural properties of the networks. In both networks, non-profit organizations – mainly sports clubs – made up the majority, while private for-profit organizations were the least represented. This is not surprising, given that cooperation between the private sector and the public or non-profit sector is generally challenging due to their different aims, values, and missions [89]. However, since the integration of private organizations in public health networks is seen as particularly beneficial due to their resources and competencies [90–92], strategies are needed to convince these actors of an engagement in sport and PA promotion. In addition to financial incentives, one approach could be to emphasize the opportunity of recruiting new members or clients through cooperation and joint projects. Another possible strategy would be to make for-profit organizations aware of the opportunity to engage in PA promotion as part of their corporate social responsibility efforts [93].

In line with a systems thinking approach, it is important to integrate change agents from various sectors into these types of networks whose primary focus is not on sports and PA promotion. Such change agents could be urban planners, transportation services, health insurance companies, or social service agencies [94]. They can have a major impact on PA-promoting structures but often do not realize that they play a crucial role [15, 63, 95, 96]. By aligning community structures, PA promotion can be approached more holistically [11]. However, too much heterogeneity among different actor groups and sectors can also be a hindrance to network effectiveness [97], which should be considered when managing and developing these networks.

The analyzed networks had a low density with a small number of realized ties. Since both were not formally established and had not yet been subject to systematic management, this is not surprising and can also be observed in other networks of this type [19, 98]. Previous studies showed that there is a need for closer cooperation and networking in the field of sport and PA promotion [15, 99]. The findings of this study provide evidence for this call for more integrated cooperation and strategic governance, as the observed networks were highly fragmented. Centralization tendencies could be identified in both networks but these were more pronounced in Network II. In both networks, the community sports administrations are among the most central network organizations, in terms of the number of cooperative ties and in terms of their function as bridging organizations. Previous studies also concluded that public and governmental sector organizations occupy a powerful position within public health networks [19, 56]. This is probably because these organizations are responsible for the distribution of financial and material resources and the coordination of cooperation is inherently one of their main tasks.

Previous research has come to mixed conclusions about what level of network size, density, and centralization is ideal. The larger the network, the greater the variety of different goals of the individual organizations [61]. This represents a challenge regarding the effectiveness of a network to solve specific problems [26]. At the same time, especially in the observed networks, there is little public funding available. Thus, by integrating more actors and by forming more relationships between existing actors, there is greater availability of resources, expertise, ideas, and mutual trust, making positive outcomes more likely [100]. It has also been shown that increased exchange and cooperation can lead to improved dissemination of information within the network [101]. For networks with a large number and diversity of actors to be effective, common network goals should be defined and documented, and their achievement should be monitored [102]. Advantages of centralized networks are that one actor or a small group of key actors organize the network activities centrally and efficiently [56]. Decentralized networks leave more room for diversity and the emergence of new ideas [57]. However, it is significantly more time-consuming for individual organizations to maintain a multitude of cooperative relationships [61], rather than to rely on a central organization to coordinate all activities. Because there is large variation in the goals and network engagement of the individual organizations surveyed, a centralized network form might therefore be more appropriate for managing cooperative activities [30].

The second aim of this study was to identify organizational and structural predictors and conditions of cooperation in interorganizational community networks of sport and PA promotion. In both networks, non-profit sector organizations cooperated with each other less frequently than would have been expected by chance. Additionally, a heterophily effect was observed among public sector organizations in Network I. Thus, cooperation in the two networks is characterized by heterophilic rather than homophilic relationships and therefore occurs in intersectoral clusters. These findings are in accordance with resource dependence theory [43], which states that organizations establish heterophilic ties with other organizations to gain access to information and resources that are not available within their own sector. Previous research concludes that homophilic relationships are more common in public health [26], yet the importance of cooperation in intersectoral clusters, in particular, is consistently emphasized. Cross-border cooperation, while more costly and difficult to manage, is thought to be more likely to help achieve structural change [10, 18, 103]. In addition, the greater diversity of available resources allows for capacity building in interorganizational networks [104]. In this respect, the heterophilic nature of cooperative ties in the studied networks can be seen as purposeful. However, it should be taken into account when managing the networks.

For-profit organizations did not show a higher level of cooperative activity, which could be attributed to the fact that they do not see any added value in increased network engagement. Furthermore, limited time and personnel resources as well as conflicting expectations regarding the objectives of cooperation could act as barriers for private-sector organizations [89]. Here, again, strategies are needed to make the benefits of network participation clear to for-profit organizations. In Network II, owning a sports facility did not lead to more cooperative ties. A reason for this could be that organizations that own a sports facility are less dependent on cooperation. This is in accordance with resource dependence theory [43].

In terms of structural predictors, cooperation in both networks was characterized by triangular structures, indicating that network organizations often cooperated in small, group-like clusters, which are inherently characterized by reciprocity, trust, and information sharing [64–66, 105]. This effect was also found in two previous studies analyzing networks of sport and PA promotion [18, 63], and is suggestive of small networks within the network. Another structural mechanism that characterized cooperation in both networks was a centralization effect. It occurs when ties within a network are not equally distributed so that a few actors have formed more relationships than others [48, 106]. These central actors, such as the community sports administrations, have a strong influence on network processes, whereupon other organizations also tend to establish cooperative ties with these central organizations, indicating a preferential attachment effect. The existence of a few important actors occupying a central position can also be observed in other informal networks or networks at an early stage of development [62, 73]. The power-law degree distribution in the observed networks with a few high-degree nodes and preferential attachment effects is similar to the organizing principles in scale-free networks as proposed by Barabási [48].

Taking the structure and mechanisms of cooperation in the observed networks into account, implications can be derived for effective network governance [30, 72]. Both networks have a low density and are centralized rather than decentralized. Because the networks were not formally established but have emerged unplanned without a strategic aim, there might be little consensus on network goals. Both networks are moderate to large in size, so the need for network-level competencies increases. However, when looking at a lower level, small triangular cooperative clusters characterized by high levels of mutual trust and interaction are also evident in both networks. Therefore, a hybrid of a lead organization- or leading group-governed network, where cooperation and information dissemination are centrally coordinated, and a participant-governed network, where the participants themselves manage the cooperation in smaller subgroups, might be the most effective governance form for both networks.

The major strength of this study is that it is one of only a few network studies in the field of public health and PA promotion [19, 56, 63] that, in addition to describing network structures, also reveals the conditions and mechanisms of network functioning through stochastic network modeling procedures. From this, a variety of starting points for the development and management of community networks of sport and PA promotion can be uncovered. In addition, using the data of networks with similar characteristics (same type of network organizations, community-based, informal networks, same cultural area, federal state, etc.) allows for the consideration of more general characteristics and mechanisms of interorganizational cooperation and a better understanding of community sport and PA networks.

Nevertheless, the study has various limitations. The data collected are self-reported, which may be inherently subject to some degree of recall bias. In addition, some organizations did not participate in the survey despite multiple reminders, so not all cooperative relationships in the network may have been captured. However, we imputed missing data by symmetrization. Since this is a secondary analysis of existing data sets, the types of cooperation surveyed are not identical in both networks. This was counteracted by dichotomizing the data and combining all cooperation types. Furthermore, the data in both networks were not collected in the same year, but with a difference of five years. However, both networks were at a similar stage (no systematic management, not formally established), so comparability is still possible. Finally, the networks analyzed represent only a snapshot of the network organizations and relationships involved at the time of the survey. Nevertheless, studies like this are still the most common approach in network research as they can provide insights into the phenomena and characteristics of a newly developing research field.

## Conclusion

This study adds to the body of knowledge on how interorganizational community networks of sport and PA promotion are structured and how they function. The analyzed networks showed various similar structural properties and mechanisms of network emergence. This knowledge allows to derive recommendations for their further development and management. Future research should focus on the evolution and dynamics of these networks in longitudinal studies to investigate whether existing structures are strengthened or weakened and which new actors get involved. To develop an overarching picture of structures and mechanisms in community networks of sports and PA providers as well as sports administrating and coordinating organizations, further analyses of this kind are needed so that findings can be consolidated. In doing so, additional organizational and structural mechanisms, different types of exchange (e.g. access to economic resources or specialized knowledge), as well as barriers to cooperation, should be considered. Finally, cooperative efforts regarding sport and PA promotion should be encouraged through greater political support and public funding to facilitate population health benefits [107].

## Electronic supplementary material

Below is the link to the electronic supplementary material.

Supplementary Material 1

## Data Availability

The datasets used and analyzed during the current study are not publicly available due to privacy reasons but are available from the corresponding author on reasonable request.

## List of abbreviations

PA: Physical activity
NRPP: National Recommendations for Physical Activity and Physical Activity Promotion
SNA: Social network analysis
CD: Degree centrality
CB: Betweenness centrality
ERGM: Exponential random graph model
GWESP: Geometrically weighted edgewise shared partner distribution
GWDegree: Geometrically weighted degree distribution
GWDSP: Geometrically weighted dyad-wise shared partner distribution
AIC: Akaike information criterion
SD: Standard deviation
