# Supplementary material for: Community networks of sport and physical activity promotion: an analysis of structural properties and conditions of cooperation
Source: BMC Public Health. 2022 Oct 26;22:1966. doi: 10.1186/s12889-022-14383-3 (PMC9608923; doi:10.1186/s12889-022-14383-3)
Supplement: Supplementary file 1 — Supplementary Material 1 [file 12889_2022_14383_MOESM1_ESM.pdf]

Community networks of sport and physical activity promotion - An analysis of structural properties and conditions of cooperation

Laura Wolbring[1], Steffen Christian Ekkehard Schmidt[1], Claudia Niessner[1], Alexander Woll[1] & Hagen Wäsche[1]

[1]Institute of Sports and Sports Science, Karlsruhe Institute of Technology, Germany

Additional file 1: Survey items for data collection

1. Network I

1.1 German version (original language)

Mit welchen Einrichtungen kooperieren Sie im Rahmen Ihres Sportangebots in Konstanz?

Bitte geben Sie an, mit welchen Einrichtungen (Sportvereine, freie Sportanbieter, sportverwaltende und -beratende Einrichtungen, Schulen, Kindergärten, Kirchen und Altenpflegeeinrichtungen) Sie im Rahmen Ihres Sportangebots kooperieren. Beziehen Sie sich dabei auf die Einrichtungen in nachfolgender Liste. Um die Liste zu betrachten, klicken Sie bitte auf den folgenden Hyperlink:

Liste der sportanbietenden und -verwaltenden Einrichtungen in Konstanz

Die Liste öffnet sich dann in einem neuen Fenster Ihres Browsers, während die Umfrage im Hintergrund geöffnet bleibt. Sie können bis zu zehn Einrichtungen nennen. Falls Sie mit mehr als zehn Einrichtungen kooperieren, nennen Sie nur die zehn wichtigsten. Beurteilen Sie für jede von Ihnen angeführte Einrichtung die Art der Kooperation:

- es findet lediglich ein Austausch von Informationen statt
- es findet eine informelle Zusammenarbeit statt (lose Zusammenarbeit um gemeinsame Ziele zu erreichen)
- es findet eine formelle Zusammenarbeit statt (enge Zusammenarbeit in einem Team um gemeinsame Ziele zu erreichen)
- es besteht eine Partnerschaft (enge Zusammenarbeit über einen längeren Zeitraum in verschiedenen Projekten)

	Austausch von Informationen	Informelle Zusammenarbeit	Formelle Zusammenarbeit	Partnerschaft
Name der Einrichtung: ☐	◻	◻	◻	◻
Name der Einrichtung: ☐	◻	◻	◻	◻
Name der Einrichtung: ☐	◻	◻	◻	◻
Name der Einrichtung: ☐	◻	◻	◻	◻
Name der Einrichtung: ☐	◻	◻	◻	◻
Name der Einrichtung: ☐	◻	◻	◻	◻

Name der Einrichtung: [ ]	☐	☐	☐	☐
Name der Einrichtung: [ ]	☐	☐	☐	☐
Name der Einrichtung: [ ]	☐	☐	☐	☐
Name der Einrichtung: [ ]	☐	☐	☐	☐

1.2 English version

With which organizations do you cooperate as part of your sports offerings in Constance?

Please indicate with which institutions (sports clubs, commercial sports providers, sports administrating and coordinating organizations, schools, kindergartens, churches, and old people’s homes) you cooperate as part of your sports offering in Constance. Refer to the organizations in the list below. To view the list, please click on the following hyperlink:

List of sports-providing and -administrating organizations in Constance.

The list will open in a new window in your browser while the survey remains open in the background. You can name up to ten organizations. If you cooperate with more than ten organizations, name only the most important ten. For each organization you name, rate the type of cooperation:

- there is only an exchange of information
- there is an informal cooperation (loose cooperation to achieve common goals)
- there is a formal cooperation (close collaboration in a team to achieve common goals)
- there is a partnership (close cooperation over a longer period of time in different projects)

	exchange of information	informal cooperation	formal cooperation	partnership
name of the organization: [ ]	☐	☐	☐	☐
name of the organization: [ ]	☐	☐	☐	☐
name of the organization: [ ]	☐	☐	☐	☐
name of the organization: [ ]	☐	☐	☐	☐
name of the organization: [ ]	☐	☐	☐	☐
name of the organization: [ ]	☐	☐	☐	☐
name of the organization: [ ]	☐	☐	☐	☐
name of the organization: [ ]	☐	☐	☐	☐
name of the organization: [ ]	☐	☐	☐	☐
name of the organization: [ ]	☐	☐	☐	☐

2. Network II

2.1 German version (original language)

2.1.1 Possession of sports facility

Verfügt Ihre Einrichtung über Sportstätten in der Oststadt?

Unter Sportstätten werden Sportanlagen (primär für den Sport geschaffen) oder Sportgelegenheiten (für andere Zwecke geschaffen, aber explizit dem Sport zur Verfügung stehende Räume, Plätze etc.) verstanden. Bitte geben Sie den Namen, Adresse, Art der Sportstätte sowie die jeweilige Größe in Quadratmetern (qm) an und ob sie für jedermann öffentlich zugänglich ist oder nicht. Berücksichtigen Sie dabei bitte, ob die Sportstätte im eigenen Besitz, zur Pacht oder in Pflege ist und machen Sie die Angaben im jeweiligen Feld.

	Name	Adresse	Art der Sportstätte	Größe in qm	Öffentlich zugänglich? (ja/nein)
im eigenen Besitz:					
im eigenen Besitz:					
im eigenen Besitz:					
zur Pacht/Miete:					
zur Pacht/Miete:					
zur Pacht/Miete:					
zur Pflege:					
zur Pflege:					
Zur Pflege:					

2.1.2 Identifying cooperation network

Mit welchen Einrichtungen kooperieren Sie im Rahmen Ihres Sportangebots in der Oststadt?

Bitte geben Sie an, mit welchen Einrichtungen (Schulen, Vereine, Kindergärten, freie Sportanbieter, Sportverwaltung, Kirchen und Altenpflegeeinrichtungen) Sie im Rahmen Ihres Sportangebots in der Oststadt kooperieren. Beziehen Sie sich dabei auf die Einrichtungen in nachfolgender Liste. Um die Liste zu betrachten, klicken Sie bitte auf den folgenden Hyperlink:

Liste der sportanbietenden und -verwaltenden Einrichtungen in oder mit Bezug zur Oststadt

Die Liste öffnet sich dann in einem neuen Fenster Ihres Browsers, während die Umfrage im Hintergrund geöffnet bleibt. Sie können bis zu zehn Einrichtungen nennen. Falls Sie mit mehr als zehn Einrichtungen kooperieren, nennen Sie nur die zehn wichtigsten. Beurteilen Sie für jede von Ihnen angeführte Einrichtung die Art der Kooperation (Mehrfachantworten sind möglich):

- es findet ein Austausch von Informationen statt
- es findet ein Austausch von Personal statt
- es findet eine Zusammenarbeit bei Sport- und Bewegungsangeboten statt
- es findet eine gemeinsame Nutzung von Sportstätten statt

	Austausch von Informationen	Austausch von Personal	Zusammenarbeit bei Angeboten	Nutzung von Sportstätten
Name der Einrichtung:	□	□	□	□
Name der Einrichtung:	□	□	□	□
Name der Einrichtung:	□	□	□	□
Name der Einrichtung:	□	□	□	□
Name der Einrichtung:	□	□	□	□
Name der Einrichtung:	□	□	□	□
Name der Einrichtung:	□	□	□	□
Name der Einrichtung:	□	□	□	□
Name der Einrichtung:	□	□	□	□
Name der Einrichtung:	□	□	□	□

2.2 English version

2.2.1 Possession of sports facility

Does your organization have sports facilities in the Oststadt?

Sports facilities are understood to be sports facilities (primarily created for sports) or sports opportunities (rooms, places, etc. created for other purposes, but explicitly available for sports). Please indicate the name, address, type of sports facility as well as the respective size in square meters (sqm) and whether it is open to the public or not. Please take into account whether the sports facility is owned, leased or maintained and provide the information in the respective field.

	name	address	type of sports facility	size in sqm	open to the public? (yes/no)
in own possession:					
in own possession:					
in own possession:					
leased:					
leased:					
leased:					
for maintenance:					
for maintenance:					
for maintenance:					

2.2.2 Identifying cooperation network

With which organizations do you cooperate as part of your sports offerings in the Oststadt?

Please indicate with which institutions (schools, sports clubs, kindergartens, commercial sports providers, sports administration, churches, and old people’s homes) you cooperate as part of your sports offering in the Oststadt. Refer to the organizations in the list below. To view the list, please click on the following hyperlink:

List of sports-providing and -administrating organizations in or related to the Oststadt.

The list will open in a new window in your browser while the survey remains open in the background. You can name up to ten organizations. If you cooperate with more than ten organizations, name only the most important ten. For each organization you name, rate the type of cooperation (multiple answers are possible):

- there is an exchange of information
- there is an exchange of personnel
- there is a cooperation in the provision of sports and physical activity programs
- there is a joint use of sports facilities

	exchange of information	exchange of personnel	cooperation on programs	use of sports facilities
name of the organization:	☐	☐	☐	☐
name of the organization:	☐	☐	☐	☐
name of the organization:	☐	☐	☐	☐
name of the organization:	☐	☐	☐	☐
name of the organization:	☐	☐	☐	☐
name of the organization:	☐	☐	☐	☐
name of the organization:	☐	☐	☐	☐
name of the organization:	☐	☐	☐	☐
name of the organization:	☐	☐	☐	☐
name of the organization:	☐	☐	☐	☐
